# Investigation of the Cellular Effects of Beta- Cyclodextrin Derivatives on Caco-2 Intestinal Epithelial Cells

**DOI:** 10.3390/pharmaceutics13020157

**Published:** 2021-01-25

**Authors:** Ágnes Rusznyák, Milo Malanga, Éva Fenyvesi, Lajos Szente, Judit Váradi, Ildikó Bácskay, Miklós Vecsernyés, Gábor Vasvári, Ádám Haimhoffer, Pálma Fehér, Zoltán Ujhelyi, Béla Nagy Jr., Zsolt Fejes, Ferenc Fenyvesi

**Affiliations:** 1 Department of Pharmaceutical Technology, Faculty of Pharmacy, University of Debrecen, Nagyerdei St. 98, H-4032 Debrecen, Hungary; rusznyak.agnes@pharm.unideb.hu (Á.R.); varadi.judit@pharm.unideb.hu (J.V.); bacskay.ildiko@pharm.unideb.hu (I.B.); vecsernyes.miklos@pharm.unideb.hu (M.V.); vasvari.gabor@pharm.unideb.hu (G.V.); haimhoffer.adam@pharm.unideb.hu (Á.H.); feher.palma@pharm.unideb.hu (P.F.); ujhelyi.zoltan@pharm.unideb.hu (Z.U.); 2 Doctoral School of Pharmaceutical Sciences, University of Debrecen, Nagyerdei St. 98, H-4032 Debrecen, Hungary; 3 Institute of Healthcare Industry, University of Debrecen, Nagyerdei St. 98, H-4032 Debrecen, Hungary; 4 Cyclolab Cyclodextrin R & D Laboratory Ltd., Illatos St. 7, H-1097 Budapest, Hungary; malanga@cyclolab.hu (M.M.); fenyvesi.e@cyclolab.hu (É.F.); szente@cyclolab.hu (L.S.); 5 Department of Laboratory Medicine, Faculty of Medicine, University of Debrecen, Nagyerdei St. 98, H-4032 Debrecen, Hungary; nagy.bela@med.unideb.hu (B.N.J.); fejes.zsolt@med.unideb.hu (Z.F.)

**Keywords:** cyclodextrins, endocystosis, NF-κB, autophagy, lysosomes

## Abstract

Cyclodextrins are widely used excipients for increasing water-solubility, delivery and bioavailability of lipophilic drugs. By using fluorescent cyclodextrin derivatives, we showed previously that cyclodextrins are able to enter Caco-2 intestinal cells by endocytosis, but the influence of different fluorescent labeling on the same cyclodextrin derivative has not been studied. The consequences of the cellular internalization of cyclodextrins have not been revealed yet either. The aims of this study were to compare the cellular internalization of fluorescein- and rhodamine-labeled (2-hydroxypropyl)-, (HPBCD) and randommethyl-β-cyclodextrins (RAMEB) and to investigate the intracellular effects of these derivatives on Caco-2 cells. Stimulation of the NF-kappa B pathway and autophagy and localization of these derivatives in lysosomes were tested. The endocytosis of these derivatives was examined by fluorescence microscopy and flow cytometry. Both fluorescein- and rhodamine-labeled derivatives entered the cells, therefore the type of the fluorescent labeling did not influence their internalization. Cyclodextrin pretreatment did not activate the translocation of the p65 subunit of the NF-kappa B heterodimer into the cell nuclei from the cytoplasm. After HPBCD or RAMEB treatment, formation of the autophagosomes did not increase compared to the control sample and at the same time these derivatives could be detected in lysosomes after internalization.

## 1. Introduction

Cyclodextrins are extensively used materials in drug formulations to increase water solubility, to engineer new delivery systems and to modulate the bioavailability of lipophilic drugs. The pharmaceutical and the food industry also use cyclodextrins to mask unpleasant flavors, reduce irritant effects or for stabilization [1,2,3]. Recently, these molecules were widely applied as active pharmaceutical ingredients and in biomedical technologies [4]. Cyclodextrins have also well-known biological effects. β-cyclodextrins can extract cholesterol from cell membranes [5], which are associated with their cytotoxicity [6]. (2-Hydroxypropyl)-β-cyclodextrin (HPBCD) can enter the cells by endocytosis and this mechanism has a major effect on the mobilization of sequestered cholesterol from the late endosomes/lysosomes of Niemann Pick mutant cells [7]. This discovery led to the clinical application of HPBCD in the treatment of Niemann-Pick disease type C [8,9].

The membrane effects and cellular uptake of cyclodextrins are well-characterized; however, the consequences of the cellular internalization of cyclodextrins have not been revealed yet. In addition, the intracellular fate and effects of cyclodextrins in different cell types are still uncovered. In this study we used Caco-2 intestinal epithelial cell line, which was reported earlier to efficiently internalize HPBCD and random methyl-β-cyclodextrin (RAMEB) [10,11]. We selected some well-known and essential cellular pathways or mechanisms to investigate the cellular effects of HPBCD and RAMEB at non-toxic concentrations. On the other hand, we reported the endocytosis of these molecules, but we have not compared the effect of different fluorescent labeling on the same cyclodextrin ring yet.

Endocytic processes form primary endocytic vesicles which deliver their content to early endosomes. During the endosomal maturation, early endosomes are converted to late endosomes, which finally can fuse with lysosomes. Transport to lysosomes via late endosomes is limited to a relatively small fractions of internalized fluid, only a specific portion is transported to lysosomes and degraded [12]. We aimed to reveal the fate of cyclodextrins after endocytosis and investigate the possibility of the transport of cyclodextrin-containing endosomes to lysosomes. For the measurement of cellular uptake of cyclodextrins, we used fluorescent cyclodextrin derivatives, which can be easily detected by fluorescent microscopy and flow-cytometry. The selected cellular pathways can reflect the impairing effect of cyclodextrins on cellular elements. No specific membrane receptor is known, which interacts with cyclodextrins [13], however the interaction of β-cyclodextrins with membrane cholesterol is well known and has several consequences [14]. The extraction of membrane cholesterol may result in the alteration of signaling pathways and mechanisms [13].

The effect of cyclodextrins on nuclear factor-κB (NF-ĸB) activation was only investigated on macrophages, where dimethyl-α-cyclodextrin antagonized the excess activation of LPS-stimulated macrophages [15]; however, the direct effect of cyclodextrins on the NF-ĸB pathway has not been investigated yet. NF-ĸB is a pleiotropic regulator of cell and viral genes, consisting of two subunits, the p50 and p65 subunits. Normally, they are found in the cytoplasm in an inactive form, but in certain cases, such as inflammatory stimuli, the complex is translocated into the nucleus and involved in the regulation of many biological processes [16,17]. The activation of the NF-ĸB pathway can be studied in Caco-2 cells by immunochemistry as we showed earlier [18,19].

Autophagy is a self-digestive process that is activated when the cell removes the damaged, aggregated proteins, or the pathologically active mitochondria, peroxisome or endoplasmic reticulum. Autophagy plays an important role in balancing energy sources [20]. Autophagy has three main types: macro-, micro- and chaperone-mediated autophagy. In the case of macro-autophagy, dysfunctional cell organelles in the cytoplasm are sequestered by the expanding phagophore, and LC3 molecules are inserted into the phagophore membrane, which becomes autophagosomes [21]. The final step of the process is the fusion with lysosomes. In our experiments we investigated the cyclodextrin’s effects on the macro-autophagy.

The aim of this study was to investigate the cellular internalization of HPBCD and RAMEB modified with different fluorophores and study the intracellular fate and effects of these derivatives on Caco-2 cells.

## 2. Materials and Methods

### 2.1. Materials

(2-hydroxypropyl)-β-cyclodextrin (HPBCD) (DS~4.5), random methyl-β-cyclodextrin (RAMEB) (DS~12) and their rhodamine (Rho) and fluorescein (FITC) labeled analogues were products of CycloLab Ltd. (Budapest, Hungary)

### 2.2. Cell Culture

The Caco-2 cell line was obtained from the European Collection of Authenticated Cell Cultures (ECACC, UK). Cells were maintained in Dulbecco’s modified Eagle’s medium (DMEM, Sigma-Aldrich Ltd., Budapest, Hungary) supplemented with 10% heat-inactivated fetal-bovine serum (Sigma-Aldrich Ltd., Budapest, Hungary), 1% non-essential amino acid (Sigma-Aldrich Ltd., Budapest, Hungary) and penicillin-streptomycin solution at 37 °C in an incubator containing 5% CO_2_. The passage number of the cells was between 37–52. The Caco-2 cell line is a colon epithelial cell line, in which the cells grow tightly together, forming a single cell layer.

### 2.3. Cytotoxicity Investigation

To determine the cytotoxicity of different concentrations of HPBCD and RAMEB Real Time Cell Analyser (RTCA DP Instrument), (XCelligence system, ACEA Biosciences Inc., San Diego, CA, USA) was used. In this experiment, 8000 cells/well were seeded on RTCA plates. After 4 days of incubation, cells were treated with HPBCD and RAMEB solutions in different concentrations. Medium was used as a negative control. Cells were incubated with the solutions for 24 h and the cell index was measured every 5 min. Values of three samples were expressed as normalized cell index calculated by the software of the instrument.

### 2.4. Intracellular Uptake of Fluorescently Labeled Cyclodextrin Derivatives on Caco-2 Cells

#### 2.4.1. Fluorescent Microscopy

In this experiment, 50,000 cells/well were seeded on round glass cover-slips placed into 12-well plates. Two days later cells were washed once with Hank’s Balanced Salt Solution (HBSS) and incubated for 30 min at 37 °C with 50 µM FITC- or Rho-HPBCD solutions. After incubation, time cells were washed four times with HBSS and fixed with 3.7% paraformaldehyde solution for 15 min at room temperature. After fixation, cells were washed four times with HBSS and cell nuclei were stained with 4′,6-diamidino-2-phenylindole (DAPI) (283 nM) for 10 min at room temperature. The cells were washed once with HBSS and the round glass cover-slips were glued to glass microscope slides. Fluorescence microscopy measurements and analyses were carried out by a Zeiss Axioscope A1 (Jena, Germany) fluorescence microscope. The following filters were used to examine the samples: DAPI: excitation G 365 nm, emission BP 445/50 nm; fluorescein: excitation BP 470/40 nm, emission BP525/50 nm; rhodamine: excitation BP 546/12 nm, emission BP 575–640 nm.

#### 2.4.2. Flow Cytometry

Flow cytometry experiments were used to confirm endocytosis. For these experiments, labeled FITC- and Rho-HPBCD and RAMEB were used. For these experiments cells were trypsinized with 0.05% trypsin-EDTA solution, washed twice with HBSS and suspended at 1 × 10^6^ cells/mL concentration. Cells were pre-incubated with different endocytosis inhibitors for 40 min at 0 °C or 37 °C, and were then incubated with 50 µM cyclodextrin solutions for 30 min at 0 °C or 37 °C. After incubation, time cells were washed three times with HBSS, and dead cells were stained by propidium iodide. Cellular fluorescence was analyzed by Becton Dickinson Beckman Coulter FC-500 flow cytometer (Pasadena, CA, USA) in three independent experiments.

### 2.5. Investigation of the NF-κB Pathway

To investigate the possible activation of the NF-κB pathway, 100,000 cells/well were seeded on round glass cover-slips placed into 12 well plates. Two or three days later, when the cells reached the appropriate confluence, cells were washed twice with HBSS and incubated for 30 min at 37 °C with 50 µM HPBCD and RAMEB or TNF-α (50 ng/mL). After the incubation time, cells were washed twice with HBSS and fixed with methanol: acetone 1:1 for 10 min at room temperature. After this incubation, time cells were washed once with HBSS and were incubated with FBS (Fetal Bovine Serum, Sigma) for 15 min at 37 °C to block the nonspecific binding sites. Cells were then washed once with HBSS and were incubated with 2 µg/mL primary anti-p65 antibody (polyclonal rabbit IgG antibody, Sigma-Aldrich) for 1 h at 37 °C. After this incubation, cells were washed four times with HBSS and incubated with 5 µg/mL secondary antibody (Alexa Fluor 488 goat anti-rabbit, Sigma-Aldrich, Budapest, Hungary) for 1 h at 37 °C in the dark. Cells were washed four times with HBSS and cell nuclei were stained with DAPI (283 nM) for 15 min at 37 °C. After this, cells were washed once with HBSS and the round glass cover-slips were glued to the slides. Fluorescence microscopy measurements and analyses were carried out by a Zeiss Axioscope A1 (Jena, Germany) fluorescent microscope. The following filters were used to examine the samples: DAPI: excitation G 365 nm, emission BP 445/50 nm; fluorescein: excitation BP 470/40 nm, emission BP525/50 nm.

### 2.6. Investigation of Autophagy

#### 2.6.1. Fluorescent Microscopy

For the qualitative analyses of autophagy, we used the LC3B Antibody Kit for Autophagy (Thermo Fisher Scientific, Budapest, Hungary). In this experiment, 100,000 cells/well were seeded on round glass cover-slips placed into 12 well plates. When cells reached the appropriate confluence, cells were washed once with HBSS and were incubated with 50 µM cyclodextrin solutions or, in the case of positive control, with 100 µM chloroquine solution for overnight at 37 °C. Cells were washed twice with HBSS and were fixed with 3.7% paraformaldehyde solution for 15 min at room temperature. After fixation, cells were washed twice with HBSS and permeabilized with 0.2% Triton X-100 solution for 15 min at room temperature. After permeabilization, cells were washed twice with HBSS and were incubated with 0.5 µg/mL primary antibody (rabbit polyclonal antibody against LC3B, Thermo Fisher Scientific, Waltham, MA, USA) for 1 h at 37 °C. After incubation time cells were washed three times with HBSS, then were incubated with 5 µg/mL secondary antibody (Alexa Fluor 488 goat anti-rabbit, Sigma-Aldrich, Budapest, Hungary) for 1 h at 37 °C in the dark. After this, cells were washed three times with HBSS and the cell nuclei were stained with DAPI (283 nM). Finally, cells were washed once with HBSS and the round glass cover-slips were glued to the slides. Fluorescent microscopy measurements and analyses were carried out by a Zeiss Axioscope A1 (Carl Zeiss Microimaging GmbH, Gottingen, Germany) fluorescent microscope. The following filters were used to examine the samples: DAPI: excitation G 365 nm, emission BP 445/50 nm; fluorescein: excitation BP 470/40 nm, emission BP525/50 nm; rhodamine: excitation BP 546/12 nm, emission BP 575–640 nm.

#### 2.6.2. Microplate Reader

For the quantitative analyses of autophagy, we used the CYTO-ID Autophagy Detection Kit (Enzo Life Sciences, Farmingdale, NY, USA). In this investigation, cells were seeded into black 96-well plates at the density of 10^4^ cell/well. After two days, when cells reached the appropriate confluence, they were washed once with PBS. After washing, cells were incubated with 50 µM HPBCD and RAMEB solutions and in the case of positive control, with 100 µM chloroquine solution overnight at 37 °C. After this, cells were washed once with PBS and were treated according to the Kit specification. Cells were incubated with a solution which contains 1 µL CYTO-ID^®^ Green Detection Reagent and 1 µL Hoechst 33,342 Nuclear Stain in 1 mL buffer for 30 min at 37 °C. After incubation time, cells were washed once with PBS. Green fluorescence intensities of the samples were measured with FLUOstar Optima microplate reader (BMG Labtech, Offenburg, Germany) at 485 nm excitation and 520 nm emission wavelengths. Hoechst 33,342 Nuclear Stain was measured at 365 nm excitation and 445 emission wavelengths. According to the instructions of the kit, green fluorescence values were normalized to the blue fluorescence values.

### 2.7. Investigation of the Lysosomes

#### 2.7.1. Fluorescence Microscopy

In this experiment, 40,000 cells/well were seeded on round glass cover-slips placed into 12-well plates. Two days later cells were washed once with HBSS and incubated for 30 min at 37 °C with 50 µM fluorescein or rhodamine labeled HPBCD and RAMEB solutions. After incubation time cells were washed three times with HBSS and incubated with the 50 nM LysoTracker^®^ fluorescent reagent for 30 min at 37 °C. After incubation cells were washed three times with HBSS and fixed with 3.7% formaldehyde solution for 15 min at room temperature. After fixation, cells were washed three times with HBSS and cell nuclei were stained with DAPI (283 nM) for 10 min at room temperature. Cells were washed once with HBSS and the round glass cover-slips were glued to the slides. Fluorescent microscopy measurements and analyses were carried out by a Zeiss Axioscope A1 (Jena, Germany) fluorescent microscope. The following filters were used to examine the samples: DAPI: excitation G 365 nm, emission BP 445/50 nm; fluorescein: excitation BP 470/40 nm, emission BP525/50 nm; rhodamine: excitation BP 546/12 nm, emission BP 575–640 nm.

#### 2.7.2. Flow Cytometry

For these experiments, FITC-HPBCD and FITC-RAMEB were used. Cells were trypsinized with 0.05% trypsin-EDTA solution, washed twice with HBSS and resuspended at 1 × 10^6^ cells/mL concentration. Cells were pre-incubated with 50 µM cyclodextrin solutions for 30 min at 37 °C and then incubated with 50 nM LysoTracker^®^ fluorescent reagent for 30 min at 37 °C. After incubation time, cells were washed two times with ice-cold HBSS and fixed with 1% PFA. Cellular fluorescence was analyzed by Guava Easy Cyte 6HT-2L flow cytometer (Merck Ltd., Darmstadt, Germany). FITC-labeled cyclodextrins were analyzed by using 488 nm excitation and 525/30nm emission wavelengths (green channel), while LysoTracker was measured at 695/50 nm.

### 2.8. Statistical Analysis

For statistical analysis SigmaStat 3.5 (Sigmastat-SPSS Inc., Chicago, IL, USA) and GraphPad Prism 5 (GraphPad-San Diego, CA, USA) softwares were used. Data are presented as means ± SD. Comparison of the groups was performed by using analysis of variance (ANOVA). Differences were considered significant at *p* < 0.05.

## 3. Results

### 3.1. Cytotoxicity

**Figure 1:**
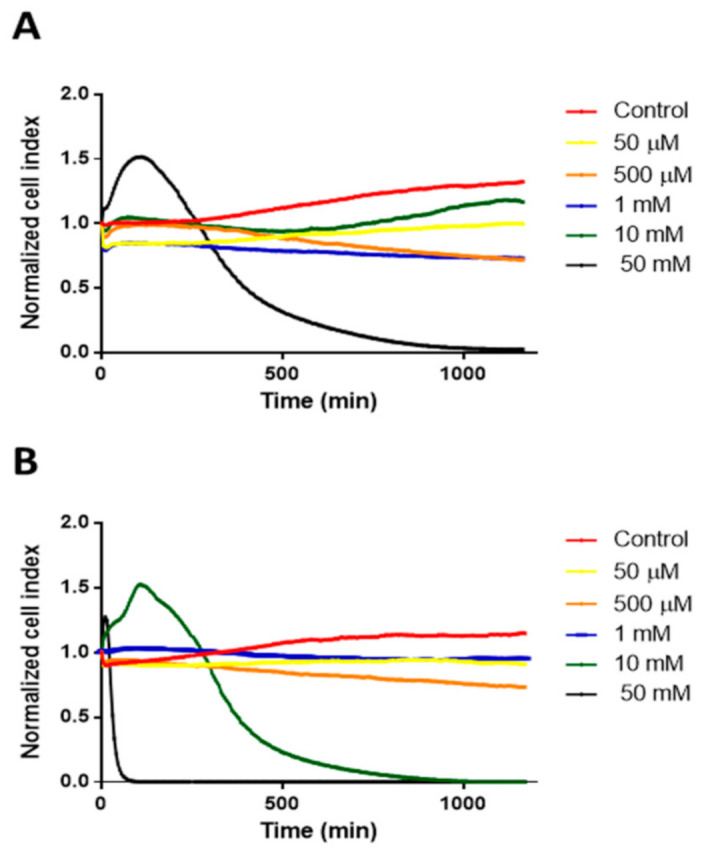
Cytotoxicity of HPBCD (**A**) and RAMEB (**B**) on Caco-2 cells as a function of time, measured by RTCA method.

The cytotoxic effects of different cyclodextrin concentrations on Caco-2 cells were investigated by RTCA method. In the case of HPBCD only the 50 mM concentration was cytotoxic, while in the case of RAMEB 10 mM concentration showed cytotoxicity (Figure 1).

**Figure 2:**
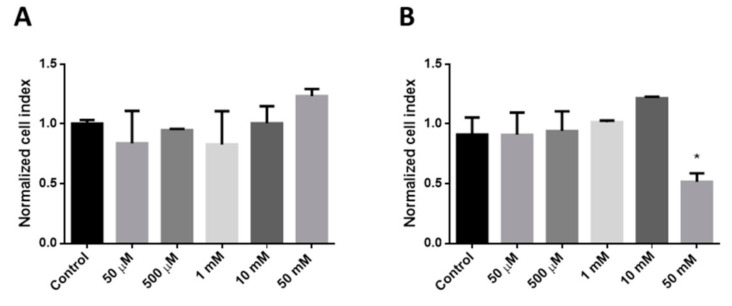
Cytotoxicity effects of HPBCD (**A**) and RAMEB (**B**) on Caco-2 cells after 30 min, measured by RTCA method. Mean ± SD are depicted (*n* = 3). * *p* < 0.05 vs. control based on ANOVA.

As we used, in our further experiments, 30 min of incubation with cyclodextrins, we analyzed the RTCA normalized cell index values at this time point (Figure 2). We found that only 50 mM RAMEB treatment showed significant toxic effects compared to the control cells after 30 min (Figure 2B). We used 50 µM non-toxic concentration of HPBCD and RAMEB with 30 min of incubation in our further experiments.

### 3.2. Investigation of the Cellular Uptake

**Figure 3:**
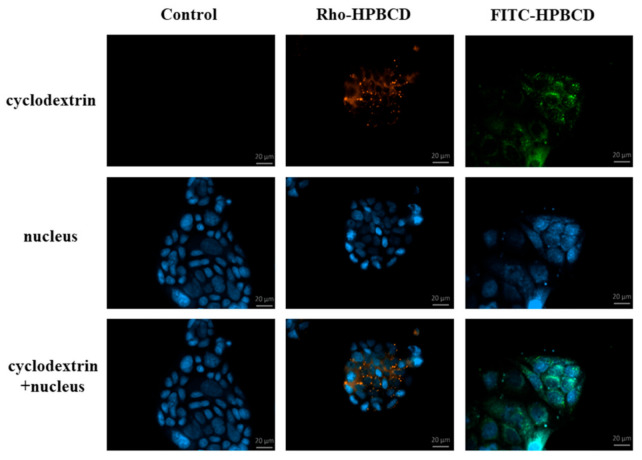
Fluorescence microscopic images of FITC-HPBCD, Rho-HPBCD and untreated Caco-2 intestinal epithelial cells. Both derivatives entered the cells and localized in small vesicles along the cell membrane and in larger granules around the nucleus. (Green-FITC-HPBCD, red-Rho-HPBCD, blue-cell nuclei).

The internalization and intracellular localization of the fluorescein or rhodamine labeled HPBCD were visualized by fluorescent microscopy using Caco-2 cells (Figure 3). Smaller and larger cyclodextrin-loaded vesicles were detected along the cell membrane and around the cell nucleus. The fluorescent labelling did not influence the endocytosis of these derivatives.

**Figure 4:**
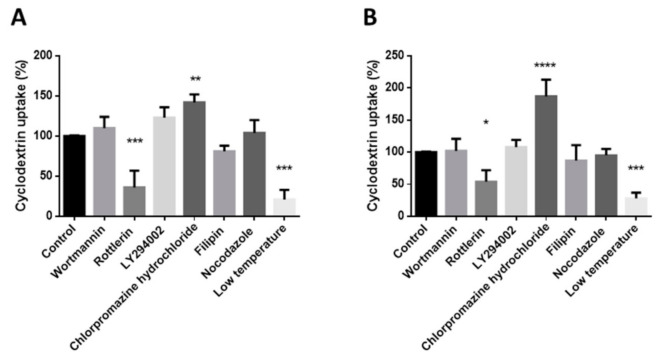
Effects of different inhibitors and cooling on the cellular uptake of FITC-HPBCD (**A**) and FITC-RAMEB (**B**) compared to the untreated control. Cooling almost perfectly inhibited the endocytosis, while rottlerin, the macropinocytosis inhibitor, significantly inhibited the endocytosis. Wortmannin and LY294002, another macropinocytosis inhibitor and filipin, which is the inhibitor of the caveola-mediated endocytosis, had no significant inhibitory effects on endocytosis. Mean ± SD are depicted (*n* = 3). * *p* < 0.05, ** *p* < 0.01, *** *p* < 0.001, **** *p* < 0.0001 vs. control based on ANOVA.

The results were confirmed by flow cytometry. For this, some samples were pre-treated with different endocytosis inhibitors and treated with 50 µM FITC-HPBCD or FITC-RAMEB at 0 °C or 37 °C. At the end of this experiment dead cells were stained with propidium iodide. Fluorescence intensity was measured by flow cytometer. We compared the fluorescence intensities of the inhibitor-treated cells to the untreated samples (100%). Both cooling and rottlerin, which is the inhibitor of macropinocytosis, significantly inhibited the cellular uptake of cyclodextrin derivatives (Figure 4). Other inhibitors had no significant inhibitory effect on the cellular uptake of cyclodextrins. Interestingly, chlorpromazine hydrochloride significantly increased both FITC-HPBCD and FITC-RAMEB cellular uptake in Caco-2 cells.

### 3.3. NF-κB Pathway

**Figure 5:**
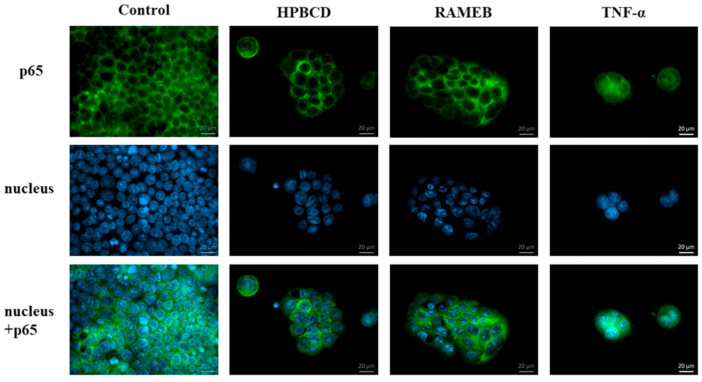
Fluorescence microscopic images of Caco-2 cells after NF-kappa B pathway investigation. HPBCD and RAMEB pre-treatment did not active the translocation of the p65 subunit of the NF-κB heterodimer into the cell nuclei from the cytoplasm, similar to the control sample. Nevertheless, TNF-α activated the translocation. (Green- p65 subunit, blue-cell nuclei).

In this experiment the effects of HPBCD and RAMEB on the NF-κB inflammatory pathway were tested. The p65 subunit of NF-κB was labeled with anti-p65 antibody and the cell nuclei were stained with Hoechst 33,342. The possible activation of NF-κB pathway by the different cyclodextrin derivatives were visualized by fluorescence microscopy (Figure 5). Cells were pre-incubated with 50 µM cyclodextrin solutions and after that primer and secondary antibody solutions. In this case, a green signal was detected only in the cytoplasm. Cyclodextrins did not activate the translocation of this subunit from the cytoplasm to the cell nucleus, so these derivatives did not activate this inflammatory pathway in the cells. In the case of positive control, cells were pre-incubated with TNF-α and a green signal was detected in the cell nuclei, indicating that TNF-α activated the NF-κB pathway.

### 3.4. Investigation of the Autophagy

**Figure 6:**
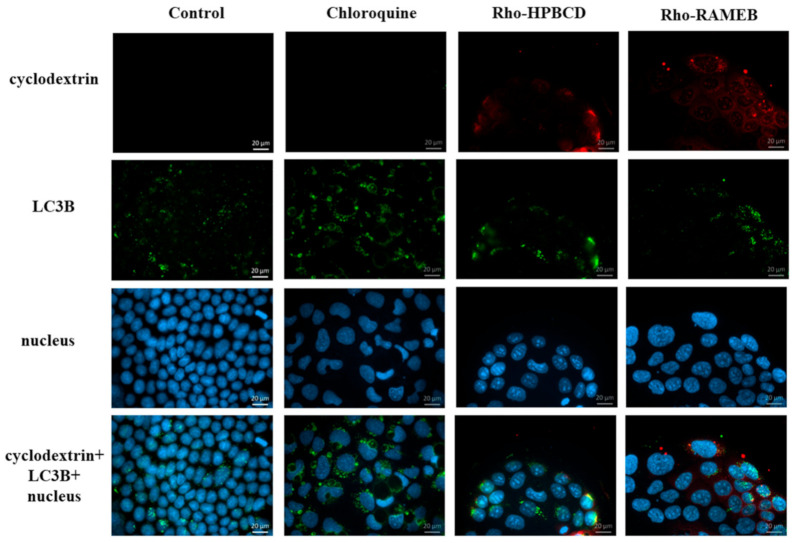
Fluorescence microscopic images of Caco-2 cells containing autophagosomes. After Rho-HPBCD and Rho-RAMEB treatments the presence of autophagosomes is similar to the control sample. After chloroquine treatment autophagosomes, labeling was more intensive.

The effects of HPBCD and RAMEB on the induction of autophagy was evaluated. First of all, we tested it qualitatively with fluorescence microscopy labeling the LC3B molecule in the autophagosome membranes with an anti-LC3B antibody while the cell nuclei were stained with Hoechst 33,342 (Figure 6). After Rho-HPBCD and Rho-RAMEB treatments, the presence of autophagosomes was detectable, similar to the control sample. At the same time, chloroquine treatment, which was the positive control, caused more intensive autophagosome formation than in the case of cyclodextrin treatment. The autophagosome formation with Rho-cyclodextrin derivatives was investigated in order to determine the localization of the cyclodextrins in the cells. We could detect limited colocalization of the green signal of LC3B and red signal of Rho-cyclodextrins in some intracellular vesicles (yellow signal) indicating that cyclodextrins were only partially localized in the autophagosomes.

**Figure 7:**
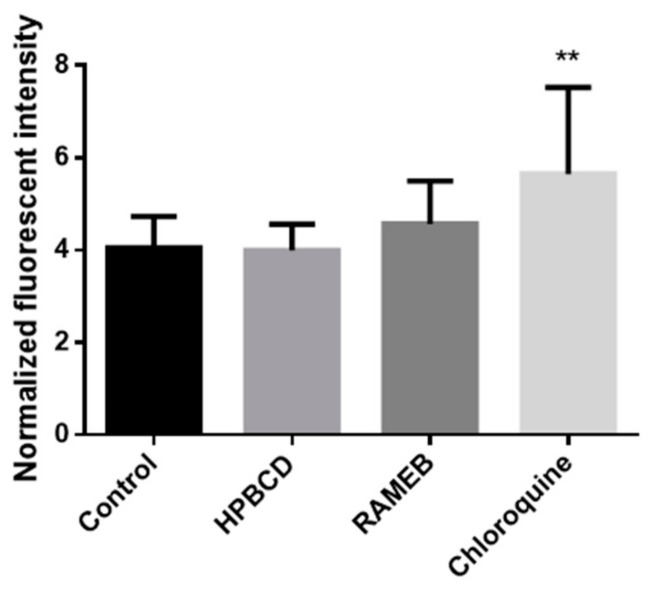
Effects of HPBCD, RAMEB and chloroquine treatments on autophagy. Cyclodextrin treatments did not increase the autophagosome formation compared to the control sample, while the chloroquine treatment significantly increased the formation. Results were normalized to the cell number and expressed as normalized fluorescence intensity. Results are expressed as means ± S.D. ** *p* < 0.01 vs. control.

We confirmed our results with quantitative experiments. The membrane of autophagosomes was stained with CYTO-ID^®^ Green Detection Reagent and the cell nuclei were stained with Hoechst 33,342. The fluorescence intensity was measured with a microplate reader. Results were normalized to cell number and expressed as a relative fluorescence intensity. Cyclodextrin treatments did not increase autophagosome formation compared to the control sample (Figure 7.). The difference between the chloroquine-treated sample and the control was significant (*p* < 0.01).

### 3.5. Investigation of the Lysosomes

**Figure 8:**
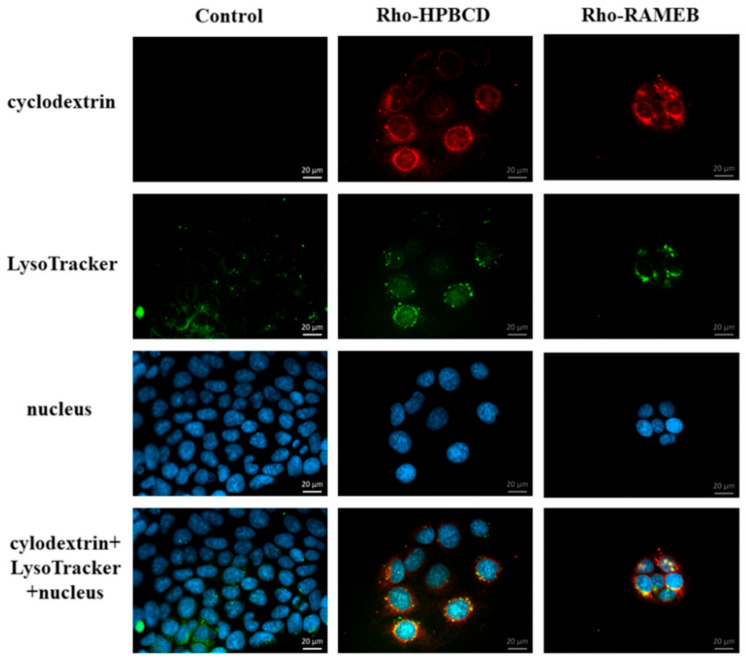
Fluorescence microscopic images of Caco-2 cells after investigation of lysosomes. Rho-HPBCD and Rho-RAMEB (**red**) are able to enter the lysosomes (**green**). Colocalization appears as yellow pixels on the images.

The effects of Rho-HPBCD and Rho-RAMEB on the lysosomes were assessed. First, a qualitative test with fluorescence microscopy was performed. After cyclodextrin treatments, lysosomes were detectable in the cytoplasm similar to the control sample (Figure 8). The green and the red signals are colocalized in many intracellular vesicles (yellow pixels), indicating that significant amount of the cyclodextrins could be detected in the lysosomes after internalization.

**Figure 9:**
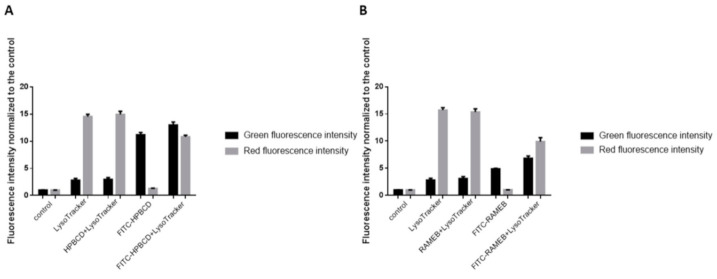
Flow cytometry analysis of lysosomes in Caco-2 cells. Mean ± SD are depicted (*n* = 4). (**A**) HPBCD and FITC-HPBCD, (**B**) RAMEB and FITC-RAMEB treatments.

We confirmed our results by flow cytometry. After unlabeled HPBCD and RAMEB treatments, the intensity of Lysotracker lysosomal staining (red fluorescence) is similar to the sample stained only with Lysotracker. After FITC-HPBCD and FITC-RAMEB treatments, the green fluorescence intensity increased, and no red fluorescence was detected. Applying both FITC-cyclodextrins and Lysotracker, the lysosomal fluorescence did not exceed the value of only Lysotracker-stained samples (Figure 9).

## 4. Discussion

In the present study, the cellular uptake and the intracellular effects of fluorescently labeled HPBCD and RAMEB were investigated.

By using fluorescent cyclodextrin derivatives, we showed previously that cyclodextrins are able to enter Caco-2 intestinal cells by endocytosis [10], but the importance of the different fluorescent labeling has not been compared yet on the same cyclodextrin derivative. Now we have confirmed our previous results, that cyclodextrins enter the cells. In the present work we compared cyclodextrin derivatives labeled with two different fluorescent moieties: the green fluorescenyl and red rhodaminyl groups attached to the cyclodextrin molecules. The type of the fluorescence labeling of HPBCD did not influence the endocytosis of these derivatives. Both fluorescein- and rhodamine-labeled derivatives entered the cells and were localized in smaller vesicles along the cell membrane and in larger granules around the nucleus. The similar chemical structure explains this: both fluorescent moieties are carboxyphenyl xanthene dyes.

The endocytosis of these cyclodextrins was found to be temperature dependent. These results were confirmed by flow cytometry measurements: cooling inhibited significantly the cellular uptake of these cyclodextrins. However, based on our results, we concluded that the mechanism of the endocytosis is the macropinocytosis, because rottlerin pretreatment reduced the fluorescence intensity significantly compared to the untreated samples. Rottlerin is one of the inhibitors of macropinocytosis. In contrast, wortmannin, another inhibitor of macropinocytosis, did not inhibit the uptake of these derivatives. This can be explained by the different mechanisms of the actions of the two inhibitors. Rottlerin is a protein kinase C inhibitor, while wortmannin and LY294002 inhibit the PI3K [22,23]. Filipin, the inhibitor of the caveola-mediated endocytosis, did not inhibit significantly the endocytosis. Nocodazole, which disrupts the microtubules, did not affect the uptake of the cyclodextrins either.

Interestingly, chlorpromazine significantly increased the endocytosis of both FITC-HPBCD and FITC-RAMEB on Caco-2 cells. Chlorpromazine is a cationic amphiphilic drug, which is applied to inhibit clathrin-coated pit formation [24]. The effect of chlorpromazine is highly cell-type dependent and in some cell types the uptake of endocytic probes were significantly increased by chlorpromazine. The possible explanation for this could be that inhibition of clathrin-coated uptake upregulates other pathways or that this amphipathic molecule affects the fluidity of the membrane in a positive way and therefore facilitates endocytosis [25]. On the other hand, chlorpromazine treatment blocked the internalization of methyl-βCD tagged with fluorescein, and the fluorescent intracellular vesicles remained in the vicinity of the plasma membrane in HeLa cells [26]. These results were obtained by fluorescence microscopy, which exactly shows the intracellular localization of endosomes. We used flow cytometry to measure the effect of inhibitors. This method measures the whole cellular fluorescence intensity and according to our results it significantly increased in Caco-2 cells after chlorpromazine treatment.

However, cyclodextrins can also facilitate membrane internalization by extracting cholesterol. Cholesterol is one of the components of the cell membrane which is crucial for maintaining the space of anionic phospholipids. Cholesterol extraction increases the negative surface charge of the cell membrane, which generates a spontaneous positive curvature [27]. Chlorpromazine-increased membrane fluidity can augment this process. It is in accordance with the higher internalization value of FITC-RAMEB after chlorpromazine treatment, because RAMEB has a higher solubilization capacity to cholesterol than HPBCD [6].

Filipin, which inhibits the caveola-mediated endocytosis, did not decrease significantly the uptake of cyclodextrins. According to these results, macropinocytosis can be the main mechanism of the cellular internalization of the fluorescent HPBCD and RAMEB derivatives in Caco-2 cells.

Autophagy is a self-digestion process which is important to degrade and recycle dysfunctional and unnecessary components. Kameyama et al. found that folate-appended methyl-β-cyclodextrin induced mitophagy, which is a specific elimination system of mitochondria [28]. In contrast to their results, in our experiments, autophagosomes were detectable in the intestinal epithelial cells after HPBCD and RAMEB treatment but these derivatives did not increase the formation of autophagosomes compared to the control sample and these cyclodextrins entered to the autophagosomes to a small extent. It indicates that cyclodextrin treatment at non-toxic, 50 µM concentrations did not cause significant impairments of the cellular components, which leads to the autophagy of the cells. On the other hand, a significant amount of cyclodextrins co-localized with lysosomes on the fluorescence microscopic images (Figure 8), but the formation of lysosomes did not increase significantly according to the flow-cytometry measurements. Neither labeled nor unlabeled cyclodextrins increased the lysosomal fluorescence. These results indicate that HPBCD and RAMEB at non-toxic concentrations enter lysosomes after endocytosis without significantly increasing the damage of cellular components, inducing autophagosome formation.

We also investigated the activation of the NF-κB pathway in Caco-2 cells by cyclodextrins at non-toxic concentrations. Translocation of the NF-κB p65 subunit into cell nuclei is a key event in inflammatory reactions. It can contribute to the epithelial barrier opening at the level of signaling pathways [29,30]. Here, we demonstrated that HPBCD and RAMEB treatment at 50 µM concentration and the consecutive cellular uptake did not cause the activation of the NF-κB pathway in Caco-2 cells.

HPBCD and RAMEB are used as pharmaceutical excipients, and HPBCD is also used as a drug for the treatment of Niemann Pick disease Type C. The cellular effects of cyclodextrins are not fully revealed, therefore we believe that the above-discussed investigations are important in the characterization and applications of these materials.

## 5. Conclusions

Even if the cellular internalization of cyclodextrins has been reported in different cell types, their further cellular effects have not been investigated yet. We tested major cellular processes, which were reported earlier to be related to the effects of cyclodextrins under different conditions. The obtained new results of this work can be summarized in the following:

1. The type of the fluorophore (FITC or Rhodamine) did not influence the endocytosis of HPBCD.
2. Macropinocytosis was the main internalization route both for HPBCD and RAMEB on Caco-2 cells.
3. HPBCD and RAMEB treatment did not induce NF-κB pathway activation on Caco-2 cells.
4. HPBCD and RAMEB treatment did not induce autophagy on Caco-2 cells.
5. Significant amount of both Rho-HPBCD and Rho-RAMEB could be detected in lysosomes after internalization in Caco-2 cells.

The novelty of this work is that we examined the effects of internalized cyclodextrins at a cellular level for the first time. According to our data, the treatment of Caco-2 cells with the non-toxic 50 µM HPBCD and RAMEB did not cause significant changes in the investigated cellular processes. The lysosomal appearance of cyclodextrins after internalization can be useful information for their further drug delivery applications.

## Data Availability

Not applicable.
